# miR-149-5p Regulates Goat Hair Follicle Stem Cell Proliferation and Apoptosis by Targeting the CMTM3/AR Axis During Superior-Quality Brush Hair Formation

**DOI:** 10.3389/fgene.2020.529757

**Published:** 2020-11-11

**Authors:** Jian Wang, Jingwen Qu, Yongjun Li, Yunkui Feng, Jinliang Ma, Liuming Zhang, Changjiang Chu, Huiru Hu, Yanhu Wang, Dejun Ji

**Affiliations:** ^1^College of Animal Science and Technology, Yangzhou University, Yangzhou, China; ^2^Key Laboratory of Animal Genetics and Molecular Breeding of Jiangsu Province, Yangzhou University, Yangzhou, China

**Keywords:** hair follicle stem cells, miR-149-5p, CMTM3, AR, proliferation, apoptosis, superior-quality brush hair formation, Yangtze River Delta white goat

## Abstract

The Yangtze River Delta white goat is a unique goat species that can produce superior quality brush hair. CKLF-like MARVEL transmembrane domain-containing 3 (CMTM3), which influences the transcriptional activity of androgen receptor (AR), was identified as a candidate gene related to superior-quality brush hair formation. CMTM3 is generally expressed at low levels, but miR-149-5p is highly expressed in the skin tissues of these goats. The mechanism by which CMTM3 regulates the proliferation and apoptosis of goat hair follicle stem cells has not been elucidated. Here, RT-qPCR, western blotting, 5-ethynyl-2′-deoxyuridine (EdU), cell cycle, apoptosis, and dual-luciferase assays were used to investigate the role and regulatory mechanism of CMTM3 and miR-149-5p. Functional studies showed that CMTM3 overexpression inhibited proliferation and induced apoptosis in cultured hair follicle stem cells, whereas silencing CMTM3 markedly facilitated cell proliferation and deterred apoptosis in cultured hair follicle stem cells. Then, using bioinformatic predictions and the aforementioned assays, including dual-luciferase assays, RT-qPCR, and western blotting, we confirmed that miR-149-5p targets CMTM3 and preliminarily investigated the interaction between CMTM3 and AR in goat hair follicle stem cells. Furthermore, miR-149-5p overexpression significantly accelerated the proliferation and attenuated the apoptosis of hair follicle stem cells. Conversely, miR-149-5p inhibition suppressed the proliferation and induced the apoptosis of hair follicle stem cells. These results reveal a miR-149-5p-related regulatory framework for the miR-149-5p/CMTM3/AR axis during superior quality brush hair formation, in which CMTM3 plays a negative role.

## Introduction

The Yangtze River Delta white goat, also known as the Haimen goat, is the exclusive goat breed in the world that can produce superior-quality brush hair. This hair is the finest raw material used for making Chinese calligraphy brushes; this breed has also been praised for this unique characteristic, which has resulted in it receiving a complimentary name: the brush hair goat (National livestock and Poultry Genetic Resources Committee, 2011). Brush hair is usually separated into three categories: Type I, inferior-quality hair; Type II, normal-quality hair; and Type III, superior-quality hair (Li and Huang, 2005). Our previous research revealed that the formation of Type III superior-quality brush hair is stimulated by androgen secretion and cold stress, which then activates and modifies the synthesis of certain proteins that participate in the formation stage of hair growth (such as fibrinogen) (Li et al., 2013; Yang et al., 2015). In previous studies, we screened and identified CKLF-like MARVEL transmembrane domain-containing 3 (CMTM3), which modulates the transcriptional activity of androgen receptor (AR), as a putative candidate gene associated with the superior-quality hair trait in Yangtze River Delta white goats (Ji et al., 2018). Upon further investigation with bioinformatic predictions and analyses, CMTM3 was identified as a target gene of miR-149-5p. The chemokine-like factor (CKLF) superfamily is a family of proteins that connects classical chemokines and transmembrane-4 superfamily factors (Han et al., 2001). The CMTM family consists of nine genes, including CKLF and CMTM1-8, and each member plays various roles in multiple biological processes (Gao et al., 2015; Zhang et al., 2016). CMTM3, also known as CKLFSF3, is closely related to AR, has a regulatory role in the male reproductive system, and is characterized by its specific leucine zipper domain and “LXXLL” (where L represents leucine and X represents any amino acid) motifs (Zhong et al., 2006).

MicroRNAs (miRNAs) are short (approximately 22 nt) non-coding RNA molecules that negatively regulate gene expression via posttranscriptional mechanisms, such as inducing target mRNA degradation or repressing translation (Wang et al., 2010). To date, many miRNAs have been identified in the skin tissues of cashmere goats (Zhang et al., 2007; Liu et al., 2012), and some other miRNAs were revealed to play critical roles in coat color genetics. Previously, several studies have reported that miRNAs, including the miR-202 (Qu et al., 2017), miR-181a (Frucht et al., 2011), and miR-let7 (Ma et al., 2018) families, are differentially expressed because their key regulators are involved in skin tissues with different hair colors and skin melanin formation in mice, goats, and sheep. Some miRNAs in skin tissue, such as miR-21 (Ahmed et al., 2011), miR-31 (Mardaryev et al., 2010), miR-214 (Ahmed et al., 2014; Du et al., 2018), miR-218-5p (Zhao et al., 2019a), and miR-320-3p (Zhao et al., 2019a), are essential for the regulation of skin and hair follicle development and regeneration. For example, miR-21 can regulate mouse hair follicle development via the BMP signaling pathway (Ahmed et al., 2011), miR-214 has been shown to suppress human hair follicle stem cell proliferation and differentiation by downregulating EZH2 with Wnt/β-catenin signaling (Du et al., 2018), and overexpression of miR-218-5p in skin fibroblast cells promotes proliferation and represses apoptosis by targeting SFPR2 (Zhao et al., 2019b). miR-149-5p and miR-149-3p comprise the miR-149 family and originate from the miR-149 precursor (based on miRbase v22.1). Recent studies have shown that miR-149-5p is a tumor-related miRNA that can play an important role in regulating cell migration by targeting GIT1 in medullary thyroid carcinoma (Ye and Chen, 2019); in addition, miR-149-5p has been confirmed as an independent prognostic indicator of clear cell renal cell carcinoma (Xie et al., 2018). Furthermore, miR-149-5p could be sponged by lncRNA SNHG8, which results in the promotion of hepatocellular carcinoma tumorigenesis and metastasis (Dong et al., 2018). However, little is known about the function of miR-149-5p in Yangtze River Delta white goats and its regulation in goat hair follicle stem cells during superior-quality hair formation.

According to our previous high-throughput sequencing results of Yangtze River Delta white goat skin tissues, CMTM3 was significantly differentially expressed between superior-quality brush hair goats and normal-quality brush hair goats (Li et al., 2013; Yang et al., 2015; Ji et al., 2018). In addition, the levels of methylated CMTM3 were notably higher while AR expression was significantly higher in superior-quality brush hair goat skin tissues than in normal-quality brush hair goat skins; this was accompanied by higher androgen levels, which are advantageous for the superior-quality brush hair trait (Wang et al., 2018). In the present study, we explored the role of CMTM3 and miR-149-5p in goat hair follicle stem cell function during the formation of superior-quality brush hair. By constructing an overexpression vector and using shRNAs to overexpress or silence endogenous CMTM3, we demonstrated that CMTM3 serves as a negative regulator of goat hair follicle stem cell proliferation and plays a positive role in apoptosis. Furthermore, we confirmed that miR-149-5p could directly target the 3′-UTR of CMTM3 mRNA, which resulted in the upregulation of AR expression. Additionally, we found that miR-149-5p serves as a positive regulator of goat hair follicle stem cells by inhibiting CMTM3, which then accelerates hair follicle stem cell proliferation and mitigates apoptosis. In total, our studies provide numerous supports for the role of miR-149-5p in regulating goat hair follicle stem cell proliferation and apoptosis and reveal a miRNA-related regulatory mechanism involving miR-149-5p, CMTM3, and AR during superior-quality brush hair formation.

## Materials and Methods

### Animal Tissue Sample Collection

Yangtze River Delta white goats, also known as brush hair goats, were obtained from the Haimen State Goat Farm (Haimen City, Jiangsu Province, China). The skin tissues from the cervical spine were collected from three normal-quality brush hair goats and three superior-quality brush hair goats (aged 4–5 months, half sibling rams). Skin tissues were immediately frozen in liquid nitrogen after harvesting. The experimental procedures used in this study were approved by the Animals Care and Use Committee of Yangzhou University.

### Expression Profiling and miRNA Prediction

Total RNA was extracted from Yangtze River Delta white goat skin tissues using TRIzol (Takara, Tokyo, Japan) and reverse-transcribed to cDNA using the PrimeScript RT reagent kit (Takara, Tokyo, Japan), which was then immediately used to perform the RT-qPCR assay of skin tissues from normal-quality and superior-quality brush hair goats. All primers used in this study were designed with Primer 5.0 software (Premier Biosoft, CA, United States) and the NCBI Primer-BLAST online website.^1^ The primers for the CMTM3 and AR genes are listed in Table 1. The GAPDH (for gene detection) gene was used as an internal control.

**TABLE 1:** Primer information for miRNA and mRNA quantitative reverse transcription.

**Gene**	**Primer name**	**Primer sequence (5′ to 3′)**	**Length**
miR-149-5p	Stem-loop RT-miR-149-5p^1^	GTCGTATCCAGTGCAGGGTCCGAGGTATTCG CACTGGATACGACGGGAGTGA	
	miR-149-5p Stem-loop-F	TCTGGCTCCGTGTCTTC	
	miR-149-5p Stem-loop-R	GTGCAGGGTCCGAGGT	
18S-rRNA ID:493779	18S-rRNA-F	GTGGTGTTGAGGAAAGCAGACA	79 bp
	18S-rRNA-R	TGATCACACGTTCCACCTCATC	
PCNA ID:102172276	PCNA-F	ATCAGCTCAAGTGGCGTGAA	213 bp
	PCNA-R	TGCCAAGGTGTCCGCATTAT	
CDK1 ID:10086361	CDK1-F	AGATTTTGGCCTTGCCAGAG	103 bp
	CDK1-R	AGCTGACCCCAGCAATACTT	
CCND2 ID:102180657	CCND2-F	GGGCAAGTTGAAATGGAA	173 bp
	CCND2-R	TCATCGACGGCGGGTAC	
CMTM3 ID:102174055	CMTM3-F	CCTCTGCTTCCTCTTTGCTGATG	129 bp
	CMTM3-R	ACGGCTGTGATGGAGATGGC	
AR ID:100860827	AR-F	CCATCTCTTCCAAGGACAGTTACC	115 bp
	AR-R	TGCTCCAATGCCTCCACACC	
Bcl2 ID:100861254	Bcl2-F	ATGTGTGTGGAGAGCGTCAA	187 bp
	Bcl2-R	CCTTCAGAGACAGCCAGGAG	
Caspase3 ID:102177031	Caspase3-F	AGGCAGACTTCTTGTACGCA	170 bp
	Caspase3-R	TTCTGTCGCTACCTTTCGGT	
Caspase9 ID:102174681	Caspase9-F	GGGGACTTCTGGTGGTTAGT	118 bp
	Caspase9-R	GAGTCAGGAGGGAGAAAGCTG	
GAPDH ID:100860872	GAPDH-F	AGGTCGGAGTGAACGGATTC	259 bp
	GAPDH-R	CCAGCATCACCCCACTTGAT	

*^1^Stem-loop RT-miR-149-5p was applied for the reverse transcription of miR-149-5p.*

miRecords^2^ and TargetScan^3^ software were employed to predict miRNAs (that could target *CMTM3*). miR-149-5p was selected based on prediction, and the specific primers for miR-149-5p used for RT-qPCR of skin tissues from normal-quality and superior-quality brush hair goats are listed in Table 1. 18S-rRNA (for miR-149-5p) was used as an internal control (Zhu and Altman, 2005; Kozera and Rapacz, 2013).

### Plasmid Construction and RNAi

The stem-loop sequence (precursor) of miR-149-5p from miRbase Release 22.1^4^ and the CDS and 3′-UTR of goat *CMTM3* from NCBI^5^ were generated and amplified from the Yangtze River Delta white goat genomes. Then, the miR-149-5p precursor sequence was cloned into the *Hin*dIII and *Xba*I sites of the overexpression vector pcDNA3.1(+) to overexpress miR-149-5p (pcDNA3.1[+]-miR-149-5p). The CDS of goat *CMTM3* was cloned into the *Not*I and *Hin*dIII sites of the pDC316-mCMV-EGFP vector to overexpress CMTM3 (CMTM3-OE). Finally, the 3′-UTR of goat *CMTM3* was cloned into the luciferase reporter vector psiCHECK-2 (Promega, Madison, WI, United States) using the *Xho*I and *Not*I restriction sites. The mutant *CMTM3* 3′-UTR luciferase reporter vector was obtained by changing the miR-149-5p binding site from GAGCCAG to GTCGGTG. The primers used for plasmid construction are shown in Table 2. ShRNAs (CMTM3-sh1, CMTM3-sh2, and CMTM3-sh3) targeting goat *CMTM3* and a shRNA scramble (sh-NC) were purchased from GenePharma (GenePharma, Suzhou, China); the sequences are shown in Table 3.

**TABLE 2:** Primers used to construct the plasmids.

**Gene**	**Primer name**	**Primer sequence (5′ to 3′)**
pcDNA3.1(+) -miR-149-5p	Pre-miR-149-5p-F	*CCCAAGCTT*TGGGAAGAGAATTGCATCCGT
	Pre-miR-149-5p-R	*GCTCTAGA*AGGACACACAGGAAGCCCT
Wild-type CMTM3	Wild-CMTM3-F	CCGCTCGAGGGCATTTCCTGTGACCCAA
	Wild-CMTM3-R	ATAAGAATGCGGCCGCGGACCACGCTGTGCTGATA
Mutant-CMTM3	Mutant-CMTM3-F	CCGCTCGAGTTGTGAATGTCGGTGAGTTCT GGACCCA
	Mutant-CMTM3-R	ATAAGAATGCGGCCGCGGACCACGCTGTGCTGATA
CMTM3-OE	CMTM3-OE-F CMTM3-OE-R	ATAAGAATGCGGCCGCATGTGGCCCCCAGACC CGGAGCC
		*CCCAAGCTT*TCTGCCTTGTCAGCTGTGGTCTC

*HindIII and *Xba*I restriction sites are italicized; *Xho*I and *Not*I restriction sites are underlined.*

**TABLE 3:** Sequence information for RNA oligonucleotides.

**Name**	**Sequence name**	**Sequence information (5′ to 3′)**
	miR-149-5p mimics	UCUGGCUCCGUGUCUUCACUCCC (sense)
		GAGUGAAGACACGGAGCCAGAUU (antisense)
	miR-149-5p NC	UUCUCCGAACGUGUCACGUTT (sense)
miR-149-5p		ACGUGACACGUUCGGAGAATT (antisense)
	miR-149-5p inhibitors	GGGAGUGAAGACACGGAGCCAGA
	miR-149-5p inhibitor NC	CAGUACUUUUGUGUAGUACAA
	CMTM3-NC	UUCUCCGAACGUGUCACGUTT (sense)
		ACGUGACACGUUCGGAGAATT (antisense)
	CMTM3-sh1	GGCCAAATTCCTCAAACAAGA (sense)
		TCTTGTTTGAGGAATTTGGCC (antisense)
CMTM3	CMTM3-sh2	GCAGAAGAAGAGAATTCCGAC (sense)
		GTCGGAATTCTCTTCTTCTGC (antisense)
	CMTM3-sh2	GCTAGGCACTTTGTCAATAAT (sense)
		ATTATTGACAAAGTGCCTAGC (antisense)
	sh-NC	GGACAGTCAGAGTGTTACAGC (sense)
		GCTGTAACACTCTGACTGTCC (antisense)

### Cell Culture and Transfection

Hair follicle stem cells from Yangtze River Delta white goats were isolated from newborn ram lamb neck skin and cultured, as described in our previous study (Wang et al., 2019). The procedures are briefly described below: (1) Skin tissues were washed with 0.9% normal saline followed by 75% ethanol with 1% penicillin-streptomycin (Invitrogen, CA, United States) three times. (2) Tissues were then rinsed with phosphate buffered saline (PBS, Solarbio, Beijing, China) three times and cut into small pieces (approximately 1 mm^3^). (3) Digestion was performed with 0.25% Trypsin-EDTA (Gibco, New York, NY, United States) at 37°C for 1.5 h. (4) After digestion, hair follicles were picked and harvested by means of a stereomicroscope (Leica, Wetzlar, Germany). (5) The harvested hair follicles were digested with 0.25% Trypsin-EDTA again at 37°C for 30 min. (6) The digested follicles were placed in DMEM-F12 (Gibco, New York, NY, United States) supplemented with 20% FBS (Gibco, New York, NY, United States) and 2% penicillin-streptomycin and were ground in a homogenizer. (7) Finally, the mixed medium was filtered through a 200-mesh cell strainer (Corning, New York, NY, United States) and cultured in 60-mm culture plates (Corning, New York, NY, United States) at 37°C. The morphological images of hair follicle stem cells and measurements of integrity are presented in Supplementary Figure S1. Hair follicle stem cells and HEK293T cells were separately cultured in 6-well plates (Corning, New York, NY, United States) with growth medium (GM) comprising DMEM-F12 supplemented with 20% FBS and 2% penicillin-streptomycin and incubated at 37°C in an atmosphere containing 5% CO_2_.

The effects of miR-149-5p on hair follicle stem cell proliferation and apoptosis were investigated by transfecting hair follicle stem cells with pcDNA3.1(+)-miR-149-5p (Table 2) and negative control (NC), miR-149-5p mimics (Mimics), single-stranded negative control (Anti-NC), and 2′-O-methylated oligonucleotides against miR-149-5p (Inhibitors) purchased from Gene Pharma (Suzhou, China) (Table 3) using Lipofectamine 3000 (Invitrogen, CA, United States) following the manufacturer’s instructions. The effects of CMTM3 on hair follicle stem cell proliferation and apoptosis were determined via transfection with CMTM3-OE (Table 2), CMTM3-NC, sh-NC, CMTM3-sh1, CMTM3-sh2, and CMTM3-sh3 (Table 3). Transfection was performed when the stem cells grew to ∼70–80% confluence. After transfection, the cells were incubated and cultured in Opti-MEM (Gibco, New York, NY, United States) for 6 h, after which the medium was replaced with fresh GM for 96 h. Then, the stem cells were collected at 24-h intervals for further experiments. All hair follicle stem cell cultures were performed at least in triplicate.

### Cell Proliferation Assay

First, hair follicle stem cells were seeded at a density of 5 × 10^5^ cells/well in 6-well plates with GM. When the stem cells grew to ∼70–80% confluence, NC, miR-149-5p mimics, anti-NC, miR-149-5p inhibitors, or pcDNA3.1(+)-miR-149-5p was separately transfected. After 6 h, the transfection medium was replaced with fresh GM for 96 h. The mRNA and protein levels of PCNA, CDK1, and CCND2 were detected by RT-qPCR and western blotting, respectively, at 24-h intervals to analyze hair follicle stem cell proliferation. Second, hair follicle stem cells were seeded at a density of 1 × 10^5^ cells/well in a 24-well plate (Corning, New York, NY, United States) and transfected with miR-149-5p oligonucleotides (Table 3) or pcDNA3.1(+)-miR-149-5p. In brief, stem cells were incubated for 2 h with serum-free medium containing 50 μM 5-ethynyl-2′-deoxyuridine (EdU) reagent from an EdU cell proliferation kit (RiboBio, Guangzhou, China) prior to immunostaining. Six independent replicate experiments were performed for each group. Fluorescent images were collected using a Leica fluorescence microscope (Leica, Wetzlar, Germany), and the imaging parameters were identical in all fluorescence microscopy images.

### Assessment of Apoptosis

First, hair follicle stem cells were cultured at a density of 5 × 10^5^ cells/well in 6-well plates with GM. After reaching ∼70–80% confluence, the stem cells were transfected with miR-149-5p oligonucleotides (Table 3) or pcDNA3.1(+)-miR-149-5p. After 6 h, the transfection medium was replaced with fresh GM for 96 h. The mRNA and protein levels of Bcl2, Caspase3, and Caspase9 were detected by RT-qPCR and western blotting, respectively, at 24-h intervals to analyze hair follicle stem cell apoptosis. Second, an Annexin V-FITC/propidium iodide (PI) staining assay was used to assess the apoptosis of hair follicle stem cells. After transfection with the abovementioned oligonucleotides or plasmids and culture for 48 h, cells subjected to different treatments were washed at least three times with 1 ml of 1 × PBS (pH = 7.4), digested and collected by trypsin, washed once more with 1 ml of 1 × PBS, and resuspended in 1 ml of 1 × binding buffer (Solarbio, Beijing, China). Afterward, the cells were treated with 5 μl of Annexin V-FITC and 10 μl of PI (Solarbio, Beijing, China) and incubated in the dark at room temperature for 10 min. Finally, the cells were analyzed using flow cytometry (FACSAria SORP, BD BioSciences, NJ, United States).

### Stem Cell RNA Isolation, Reverse Transcription PCR (RT-PCR), and Real-Time Quantitative PCR (RT-q-PCR)

Total RNA was extracted from hair follicle stem cells cultured *in vitro* using a TRIzol kit (Takara, Tokyo, Japan). For gene quantification, 1 μl of total RNA (1000 ng/μl) was reverse-transcribed into cDNA using the PrimeScript RT kit (Takara, Tokyo, Japan) and then quantified on an ABI 7500/7500-Fast Real-Time PCR System (Applied Biosystems, CA, United States) with TB Green II Master Mix Reagent Kit (Takara, Tokyo, Japan). For miR-149-5p quantification, 1 μl of total RNA (1000 ng/μl) and a miR-149-5p stem-loop primer or a pair of miR-149-5p-specific primers (Table 1) were used for miR-149-5p RT-PCR and RT-qPCR, respectively. GAPDH (for gene detection) and 18S-rRNA (for miR-149-5p) were selected as internal normalization controls. The reaction conditions were as follows: 95°C for 30 s (initial denaturation), 40 cycles of 95°C for 10 s (denaturation) and then 60°C for 1 min (annealing), and an elevated optimum temperature for 5 min (final extension). The relative gene expression level was calculated using the 2^–ΔΔCt^ method (Arocho et al., 2006; Adnan et al., 2011).

### Western Blotting

Total cellular protein was extracted from each treatment group using RIPA lysis buffer (Solarbio, Beijing, China) supplemented with 1% PMSF (Solarbio, Beijing, China). Cell protein fractions were prepared and collected by centrifugation (13 000 × g, 4°C, 5 min) and then quantified using a BCA protein assay kit (Solarbio, Beijing, China). For detection, 20 μg of cellular proteins was separated via SDS-polyacrylamide gel electrophoresis with 8% or 10% gels and subsequently transferred to polyvinylidene fluoride (PVDF) membranes (Immobilon, Darmstadt, Germany), which were then blocked with 5% skim milk (Sangon Biotech, Shanghai, China) for 2 h at room temperature. Subsequently, the blocked PVDF membranes were incubated overnight at 4°C with primary antibodies against PCNA (MW: 29 kDa, Abcam, Cambridge, United Kingdom, 1:1000 dilution), CDK1 (MW: 34 kDa, Abcam, Cambridge, United Kingdom, 1:1000 dilution), CCND2 (MW: 33 kDa, Abcam, Cambridge, United Kingdom, 1:1000 dilution), Bcl2 (MW: 26 kDa, Proteintech, Rosemont, IL, United States, 1:1000 dilution), Caspase3 (MW: 32 kDa, Proteintech, Rosemont, IL, United States, 1:1000 dilution), Caspase9 (MW: 46 kDa, Proteintech, Rosemont, IL, United States, 1:1000 dilution), AR (MW: 68 kDa, Abcam, Cambridge, United Kingdom, 1:1000 dilution), CMTM3 (MW: 20 kDa, Bioss, Beijing, China, 1:1000 dilution) and β-actin (MW: 42 kDa, Abcam, Cambridge, United Kingdom, 1:500 dilution). Then, the membranes were washed with 1 × Tris-buffered saline buffer supplemented with Tween 20 (TBST) (Solarbio, Beijing, China) before they were incubated with horseradish peroxidase-conjugated secondary antibodies, including goat-specific anti-rabbit IgG and rabbit-specific anti-goat IgG (Bioworld, Nanjing, China, 1:5000 dilution), for 2 h. Protein bands were visualized using Super-enhanced ECL Reagent (Biosharp, Hefei, China) and then analyzed on a FluorChem FC3 system (Protein-Simple, CA, United States). Finally, the band intensities on the images were analyzed using ImageJ software.

### Cell Cycle Assay

A cell cycle PI staining assay was performed to determine the effects of miR-149-5p oligonucleotides, pcDNA3.1(+)-miR-149-5p, CMTM3-OE, and CMTM3-shRNAs on the different phases of the cell cycle in hair follicle stem cells. After cells were transfected for 48 h, they were collected in 6-well plates and centrifuged at 1500 rpm/min for 5 min. The supernatant was discarded after centrifugation, and the pelleted cells were washed once with 1 ml of precooled 1 × PBS. Afterward, cells were incubated with 1 ml of precooled 70% ethyl-alcohol at 4°C for 12 h. Subsequently, the cells were resuspended in 500 μl of PI staining buffer (Beyotime, Shanghai, China) and incubated at 37°C for 30 min in the dark. Then, the cell suspensions were subjected to flow cytometry analysis (FACSAria SORP, BD BioSciences, NJ, United States). ModFit LT^TM^ software (Verity Software House, Topsham, ME, United States) was used to create and analyze the cell cycle histograms.

### Dual-Luciferase Assay

HEK293T cells were cultured in 24-well Corning plates in DMEM-F12 supplemented with 20% FBS and 2% penicillin-streptomycin. Transfection was performed when cells grew to ∼70–80% confluence. The miR-149-5p oligos comprising miR-149-5p NC, miR-149-5p mimics, miR-149-5p anti-NC, and miR-149-5p inhibitors were cotransfected with psiCHECK-2 goat *CMTM3-*3′-UTR (wild-type *CMTM3*-3′-UTR) or psiCHECK-2 goat CMTM3-mut-3′-UTR (mutant-*CMTM3*-3′-UTR) into HEK293T cells with Lipofectamine 3000. Additionally, pcDNA3.1(+)-miR-149-5p was cotransfected with wild-type *CMTM3*-3′-UTR or mutant *CMTM3*-3′-UTR into HEK293T cells with Lipofectamine 3000. Forty-eight hours after transfection, a Dual-Luciferase Reporter Assay kit (TransGen, Beijing, China) was used to quantify the relative luciferase activity in each well according to the manufacturer’s protocols. Firefly and Renilla luciferase activities were assessed on a BioTek Synergy 2 Multimode Microplate Reader (BioTek, VT, United States). The firefly luciferase activity was normalized to the Renilla luciferase activity.

### Statistical Analysis

All data produced in this study are shown as the mean ± standard error of the mean (SEM) and are based on at least three or six independent biological replicates for each assay. One-way ANOVA was performed in SPSS v24 software (IBM, Armonk, NY, United States) to analyze miR-149-5p, CMTM3, and AR expression levels in hair follicle stem cells at 24 h intervals. Independent-samples *t*-tests were performed in SPSS v24 and Origin 7.5 software (OriginLab, MA, United States) to analyze and compare two different treatment groups (such as normal-quality vs. superior-quality; NC vs. Mimics; Anti-NC vs. Inhibitors; CMTM3-NC vs. CMTM3-OE; etc.). *P*-values < 0.05 were considered to be significant. ^∗^*P* < 0.05 and ^∗∗^*P* < 0.01.

## Results

### miR-149-5p, CMTM3, and AR Expression in Yangtze River Delta White Goat Skin Tissue and Goat Hair Follicle Stem Cells

**FIGURE 1:**
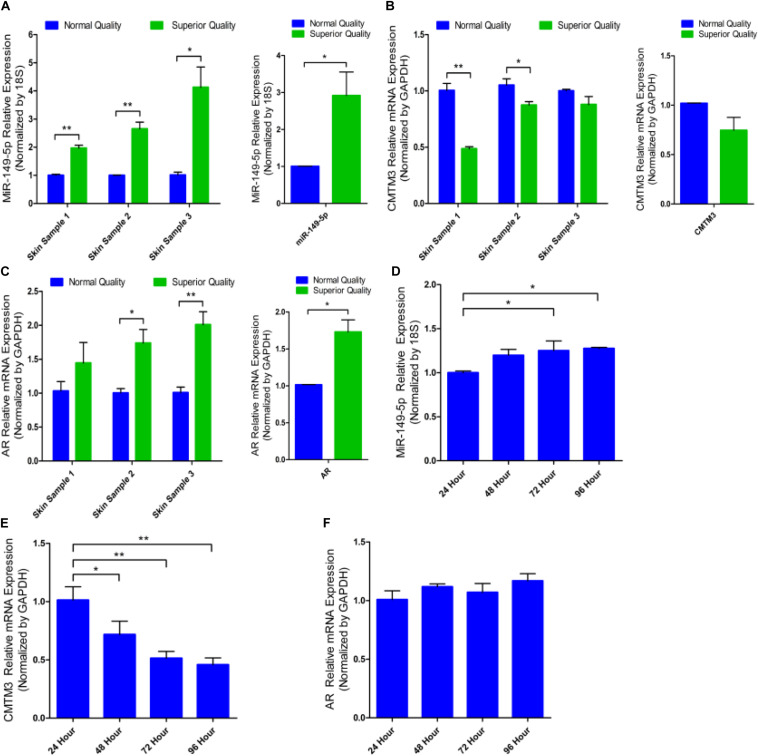
miR-149-5p, CMTM3, and AR expression in skin tissues and cultured hair follicle stem cells from brush hair goats. The differences in miR-149-5p **(A)**, CMTM3 gene **(B)**, and AR gene **(C)** expression in skin tissues between three normal-quality brush hair goats and three superior-quality brush hair goats. Relative expression of miR-149-5p **(D)**, CMTM3 **(E)**, and AR **(F)** at 24 h, 48 h, 72 h, and 96 h were examined in GM by RT-qPCR of hair follicle stem cells. The results from each group are shown as the mean ± SEM of three independent replicates. One-way ANOVA and independent-samples *t*-tests were used for statistical analysis. Asterisks indicate significant differences. No asterisk means *P* > 0.05, *P < 0.05, and **P < 0.01.

RT-qPCR analysis of miR-149-5p, CMTM3, and AR mRNA levels was performed on skin tissues from three normal-quality brush hair goats and three superior-quality brush hair goats to investigate differences in expression and explore the potential function of miR-149-5p and CMTM3. Difference analysis showed that miR-149-5p expression was higher in skin tissues from superior-quality brush hair goats than in those from normal-quality brush hair goats (*P* < 0.05) (Figure 1A). By contrast, CMTM3 expression was lower in superior-quality brush hair goats than that in normal-quality brush hair goats, but this difference was not significant (*P* > 0.05) (Figure 1B). AR expression was also higher in superior-quality brush hair goat skin samples than that in normal-quality brush hair goat skin samples (*P* < 0.05) (Figure 1C) because superior-quality brush hair is only formed in ram goats (aged 4–5 months), and this expression trend is consistent with the RNA-seq results of AR expression in goat hair follicle stem cells after CMTM3 interference (unpublished data). We also investigated miR-149-5p, CMTM3, and AR expression levels in untreated hair follicle stem cells in GM at 24 h, 48 h, 72 h, and 96 h. We observed that miR-149-5p expression levels were increased in untreated hair follicle stem cells at 24 h, 48 h, 72 h, and 96 h (at 48 h, *P* > 0.05; at 72 h and 96 h, *P* < 0.05) (Figure 1D); the mRNA level of CMTM3 was significantly decreased in untreated hair follicle stem cells at 24 h, 48 h, 72 h, and 96 h (at 48 h, *P* < 0.05; at 72 h and 96 h, *P* < 0.01) (Figure 1E); and the mRNA level of AR was increased over time in untreated hair follicle stem cells, but this change was not significant at 24 h, 48 h, 72 h, and 96 h (at 48 h, 72 h, and 96 h, *P* > 0.05) (Figure 1F). These results suggested that miR-149-5p and CMTM3 play antagonistic roles in hair follicle stem cells, and we hypothesized that the miR-149-5p/CMTM3/AR axis regulates the process of superior-quality brush hair formation. Thus, we further investigated miR-149-5p by assessing the individual effects of synthetic miR-149-5p mimics, miR-149-5p inhibitors, and a constructed overexpression vector (pcDNA3.1[+]-miR-149-5p plasmid) on hair follicle stem cells. The delivery of miR-149-5p duplexes was remarkably effective, increasing or reducing miR-149-5p levels by > 30-fold, respectively, in hair follicle stem cells (*P* < 0.01) (Supplementary Figures S2A,B).

### CMTM3 Inhibits Proliferation and Promotes Apoptosis of Goat Hair Follicle Stem Cells

**FIGURE 2:**
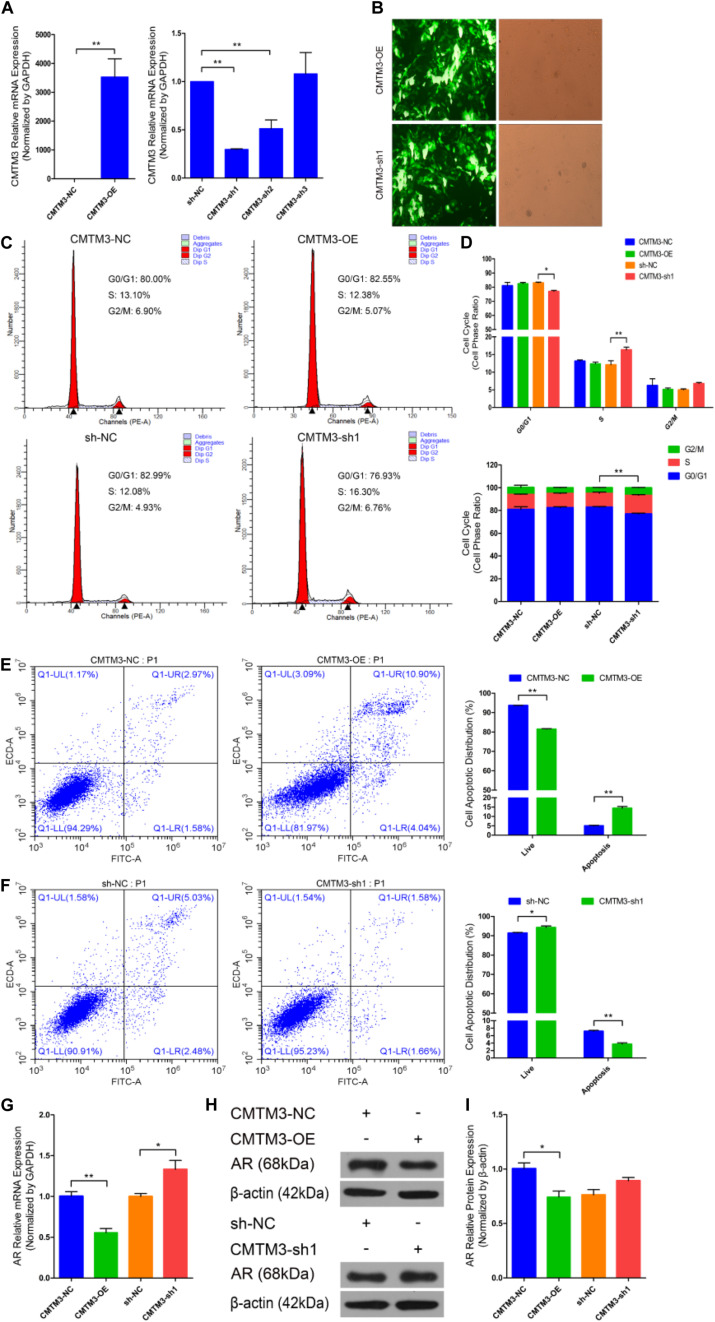
Role of CMTM3 in goat hair follicle stem cell proliferation and apoptosis. **(A)** CMTM3 expression was measured 24 h after transfection with CMTM3-NC, CMTM3-OE, sh-NC, CMTM3-sh1, CMTM3-sh2, and CMTM3-sh3 in GM. **(B)** Images of EGFP in hair follicle stem cells at 24 h after transfection with CMTM3-OE and CMTM3-sh1 in GM. Hair follicle stem cells were transfected with CMTM3-NC, CMTM3-OE, sh-NC, and CMTM3-sh1 in GM, and cell phases were analyzed 24 h after transfection by flow cytometry **(C)** and counted **(D)**. **(E)** Hair follicle stem cells were transfected with CMTM3-NC or CMTM3-OE in GM, and cell apoptosis was analyzed 48 h after transfection by Annexin V-FITC/PI binding followed by flow cytometry. **(F)** Hair follicle stem cells were transfected with sh-NC or CMTM3-sh1 in GM, and cell apoptosis was analyzed 48 h after transfection by Annexin V-FITC/PI binding followed by flow cytometry. **(G)** AR expression was measured after transfection with CMTM3-NC, CMTM3-OE, sh-NC, and CMTM3-sh1 in GM. **(H,I)** AR (1:1000 dilution) protein expression was examined after transfection with CMTM3-NC, CMTM3-OE, sh-NC, and CMTM3-sh1 in GM. The results from each group are shown as the mean ± SEM of three independent replicates. Independent-samples *t*-tests were used for statistical analysis. Asterisks indicate significant differences. No asterisk means *P* > 0.05, *P < 0.05, and **P < 0.01.

We first identified the role of CMTM3 during hair follicle stem cell proliferation and apoptosis by overexpressing or silencing endogenous CMTM3 in these cells using the pDC316-mCMV-EGFP-CMTM3 vector (CMTM3-OE) or shRNA targeting CMTM3, respectively. CMTM3 mRNA expression was notably increased in the CMTM3-OE group and extremely reduced in the CMTM3-sh1 group (*P* < 0.01) (Figure 2A). Fluorescent images of EGFP in hair follicle stem cells indicated that CMTM3-OE and CMTM3-sh1 were successfully transfected into the stem cells (Figure 2B). These results showed that CMTM3 expression (at the mRNA level) was effectively overexpressed or suppressed by CMTM3-OE and CMTM3-sh1, respectively, in hair follicle stem cells.

Next, cell cycle and Annexin V-FITC/PI staining assays were performed to investigate the role of CMTM3 during the proliferation and apoptosis of hair follicle stem cells. Cell cycle analysis revealed that CMTM3 overexpression via transfection with CMTM3-OE decreased the number of hair follicle stem cells at S-phase (from 13.10% down to 12.38%, *P* > 0.05) and increased the proportion of cells in G0/G1-phase (from 80.00% up to 82.55%, *P* > 0.05). In contrast, knocking down CMTM3 via transfection with CMTM3-sh1 significantly increased the number of hair follicle stem cells at the S-phase (from 12.08% up to 16.30%, *P* < 0.01) and notably decreased the proportion of the cells in G0/G1-phase (from 82.99% down to 76.93%, *P* < 0.05) (Figures 2C,D). The Annexin V-FITC/PI staining assay showed that CMTM3 overexpression clearly accelerated apoptosis in hair follicle stem cells and strongly increased the apoptotic cell proportion (*P* < 0.01) (Figure 2E), while decreased CMTM3 expression protected hair follicle stem cells from apoptosis and profoundly decreased the proportion of apoptotic cells (*P* < 0.01) (Figure 2F). These results showed that CMTM3 overexpression functions in a manner consistent with that of miR-149-5p inhibition on hair follicle stem cell proliferation and apoptosis. Conversely, the effect of CMTM3 inhibition on hair follicle stem cell proliferation and apoptosis was consistent with that of miR-149-5p overexpression. Moreover, RT-qPCR and western blotting were used to assess AR mRNA and protein expression levels, respectively, in hair follicle stem cells with CMTM3 overexpression or knockdown. The results showed that AR mRNA and protein expression levels were markedly decreased in hair follicle stem cells transfected with CMTM3-OE (*P* < 0.05) but enhanced (*P* < 0.05 and *P* > 0.05, respectively) in cells transfected with CMTM3-sh1 (Figures 2G–I). Taken together, these results indicated that overexpressing endogenous CMTM3 repressed hair follicle stem cell proliferation and induced apoptosis, whereas silencing endogenous CMTM3 expression accelerated hair follicle stem cell proliferation and inhibited apoptosis. In addition, AR expression was regulated by CMTM3 overexpression or inhibition in hair follicle stem cells.

### miR-149-5p Directly Targets the 3′-UTR of Goat CMTM3 and Upregulates AR Expression

**FIGURE 3:**
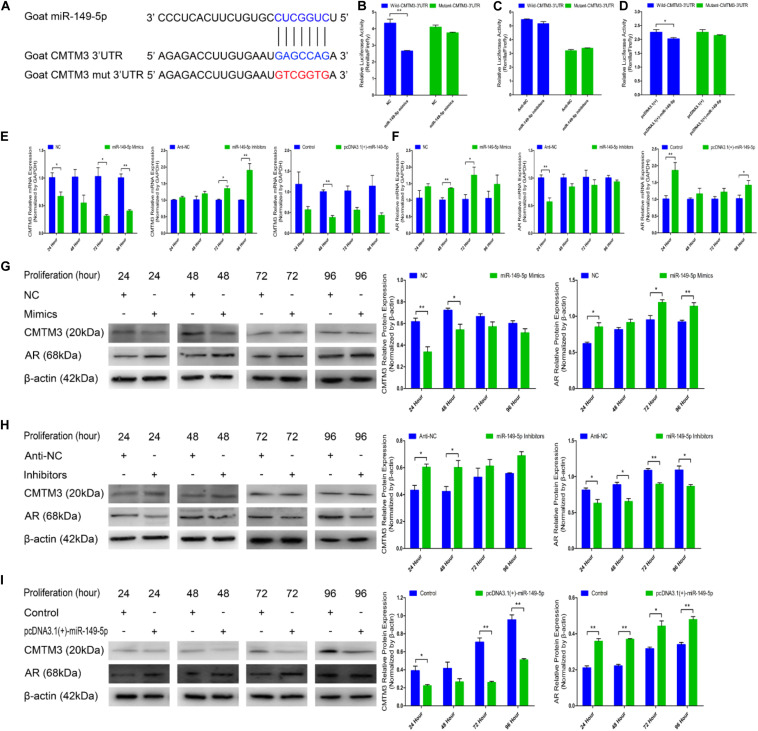
miR-149-5p suppresses CMTM3 expression and accelerates AR expression by directly targeting the 3′-UTR of CMTM3. **(A)** The predicted binding site (blue) and mutated site (red) of miR-149-5p in the 3′-UTR of goat CMTM3. **(B–D)** Dual-luciferase activity assay of the wild-type or mutant 3′-UTR of CMTM3. NC or miR-149-5p mimics were cotransfected with wild-type or mutant CMTM3 3′-UTR luciferase reporters in HEK293T cells **(B)**. Anti-NC or miR-149-5p inhibitors were cotransfected with wild-type or mutant CMTM3 3′-UTR luciferase reporters in HEK293T cells **(C)**. pcDNA3.1(+) or pcDNA3.1(+)-miR-149-5p was cotransfected with wild-type or mutant CMTM3 3′-UTR luciferase reporters in HEK293T cells **(D)**. **(E)** CMTM3 mRNA expression during hair follicle stem cell proliferation after transfection with NC, miR-149-5p mimics, anti-NC, miR-149-5p inhibitors or pcDNA3.1(+)-miR-149-5p as evidenced by RT-qPCR; total RNA was harvested at 24 h, 48 h, 72 h, and 96 h after transfection. **(F)** AR mRNA expression during hair follicle stem cell proliferation after transfection with NC, miR-149-5p mimics, anti-NC, miR-149-5p inhibitors or pcDNA3.1(+)-miR-149-5p was evidenced by RT-qPCR; total RNA was harvested at 24 h, 48 h, 72 h, and 96 h after transfection. **(G–I)** CMTM3 (1:1000 dilution) and AR (1:1000 dilution) protein expression levels were examined after transfection with NC, miR-149-5p mimics **(G)**, anti-NC, miR-149-5p inhibitors **(H)**, and pcDNA3.1(+)-149-5p **(I)** in GM at 24 h, 48 h, 72 h, and 96 h. The results from each group are shown as the mean ± SEM of three independent replicates. Independent-samples *t*-tests were used for statistical analysis. Asterisks indicate significant differences. No asterisk means *P* > 0.05, *P < 0.05, and **P < 0.01.

We explored the mechanisms of the effects of CMTM3 and miR-149-5p using bioinformatic databases (*TargetScan;^6^ miRecords;^7^ David^8^*); CMTM3 (the candidate gene involved in the growth and formation of superior-quality brush hair) was selected among the potential target genes of miR-149-5p. We found that the 3′-UTR of goat CMTM3 mRNA contained a highly conserved binding site capable of complementing the miR-149-5p seed sequence in the databases (Figure 3A).

In the preliminary experiments, we observed a notable reduction in luciferase activity in only the pcDNA3.1(+)-miR-149-5p treatment in the pre-dual-luciferase assay (*P* < 0.05), and luciferase activity was non-significantly decreased in the pre-dual-luciferase assay in the pcDNA3.1(+)-miR-365-3p, pcDNA3.1(+)-miR-23a-3p, and pcDNA3.1(+)-miR-23b-3p treatments compared with the control (*P* > 0.05) (Supplementary Figure S3). We further verified whether CMTM3 is a precise target gene of miR-149-5p by introducing NC, miR-149-5p mimics, anti-NC, miR-149-5p inhibitors, a pcDNA3.1(+) null-plasmid, and pcDNA3.1(+)-miR-149-5p into HEK-293T cells and hair follicle stem cells cultured in GM. We constructed psiCHECK-2 double-luciferase reporters that included a separate fragment of the wild-type or mutant goat *CMTM3* 3′-UTR (Figure 3A). The wild-type- or mutant-*CMTM3*-3′-UTR plasmids were cotransfected with miR-149-5p oligos, pcDNA3.1(+) null-plasmid, or pcDNA3.1(+)-miR-149-5p into HEK-293T cells. The results showed that compared with the NC and wild-type *CMTM3*-3′-UTR plasmid cotransfection group, the miR-149-5p mimics and wild-type *CMTM3*-3′-UTR plasmid cotransfection group showed significantly reduced luciferase activity, whereas no noticeable reduction in luciferase activity was observed with the miR-149-5p mimics and mutant-*CMTM3*-3′-UTR plasmid cotransfection group compared with the NC and mutant-*CMTM3*-3′-UTR plasmid cotransfection group (*P* < 0.01) (Figure 3B). Additionally, no noticeable reduction in luciferase activity was observed in cells cotransfected with the wild-type- or mutant-*CMTM3*-3′-UTR plasmid and either anti-NC or miR-149-5p inhibitors (*P* > 0.05) (Figure 3C). Similarly, a clear reduction in luciferase activity was obtained in the pcDNA3.1(+)-miR-149-5p and wild-type *CMTM3*-3′-UTR plasmid cotransfection groups, which is consistent with the miR-149-5p mimics-treated groups (*P* < 0.05) (Figure 3D). These results preliminarily confirmed that miR-149-5p directly targets the 3′-UTR of goat *CMTM3*. Furthermore, CMTM3 mRNA and protein expression was measured to verify the relationship between CMTM3 and miR-149-5p, and AR mRNA and protein expression was preliminarily assessed to investigate the correlation between CMTM3 and AR in hair follicle stem cells. The results indicated that miR-149-5p overexpression repressed CMTM3 expression (at the mRNA and protein levels) at 24 h, 48 h, 72 h, and 96 h in cells cultured in GM alone but that this difference did not reach a significant level at some time points (such as CMTM3 mRNA expression at 48 h, *P* > 0.05) and was accompanied by the upregulation of AR mRNA and protein expression, which was not significant at some time points (such as AR mRNA expression at 24 h and 48 h, *P* > 0.05). Conversely, miR-149-5p inhibition increased CMTM3 mRNA (Figure 3E) and protein expression (Figures 3G–I) and downregulated AR mRNA (Figure 3F) and protein expression (Figures 3G–I); however, no level of significance was reached at some time points (such as CMTM3 mRNA expression at 24 h and 48 h, AR mRNA expression at 48 h to 96 h, *P* > 0.05). Taken together, these results indicated that miR-149-5p inhibits goat CMTM3 mRNA and protein expression in hair follicle stem cells by directly targeting the CMTM3 mRNA 3′-UTR region. The inhibition of CMTM3 was accompanied by the upregulation of AR mRNA and protein expression.

### miR-149-5p Positively Regulates Goat Hair Follicle Stem Cell Proliferation

**FIGURE 4:**
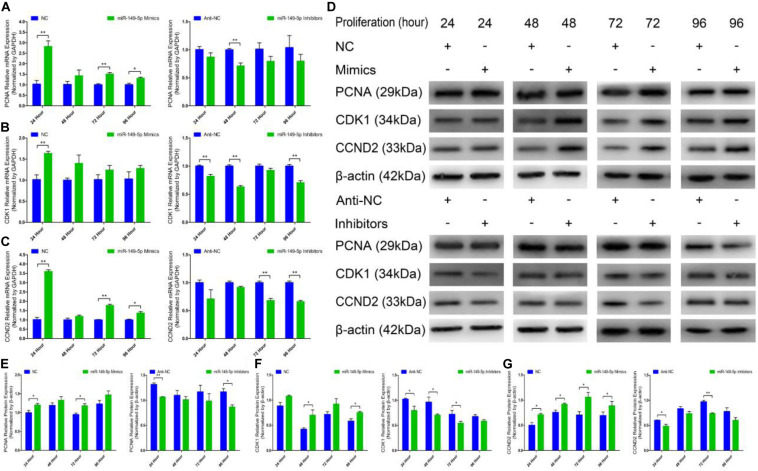
Expression of proliferation marker genes and regulation of miR-149-5p during the proliferation of goat hair follicle stem cells. PCNA **(A)**, CDK1 **(B)**, and CCND2 **(C)** mRNA expression at 24 h, 48 h, 72 h, and 96 h after transfection with NC, miR-149-5p mimics, anti-NC, and miR-149-5p inhibitors in GM as determined by RT-qPCR. **(D)** Protein expression of PCNA (1:1000 dilution), CDK1 (1:1000 dilution), and CCND2 (1:1000 dilution) was measured at 24 h, 48 h, 72 h, and 96 h after transfection with NC, miR-149-5p mimics, anti-NC, and miR-149-5p inhibitors in GM. β-actin (1:500 dilution) was used as an internal control. **(E)** Relative PCNA protein expression after transfection with NC, miR-149-5p mimics, anti-NC, and miR-149-5p inhibitors in GM at 24 h, 48 h, 72 h, and 96 h. **(F)** Relative CDK1 protein expression after transfection with NC, miR-149-5p mimics, anti-NC, and miR-149-5p inhibitors in GM at 24 h, 48 h, 72 h, and 96 h. **(G)** Relative CCND2 protein expression after transfection with NC, miR-149-5p mimics, anti-NC, and miR-149-5p inhibitors in GM at 24 h, 48 h, 72 h, and 96 h. The results from each group are shown as the mean ± SEM of three independent replicates. Independent-samples *t*-tests were used for statistical analysis. Asterisks indicate significant differences. No asterisk means *P* > 0.05, *P < 0.05, and **P < 0.01.

**FIGURE 5:**
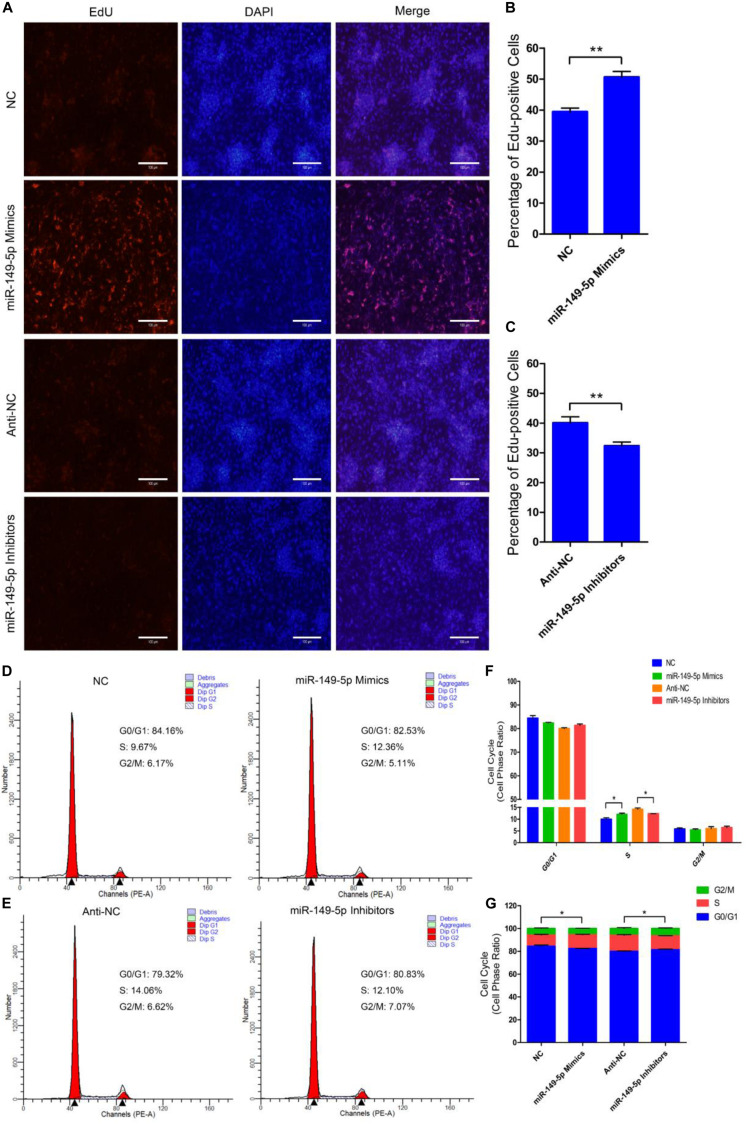
miR-149-5p accelerates goat hair follicle stem cell proliferation. **(A)** Representative images of the EdU assay of hair follicle stem cells at 24 h after transfection with NC, miR-149-5p mimics, anti-NC, and miR-149-5p inhibitors in GM. Bars, 100 μm. **(B,C)** Quantification of EdU-positive cells (*n* = 6). The ratio of EdU-positive cells was calculated as (EdU-positive cells/Hoechst stained cells) × 100%. **(D–G)** Hair follicle stem cells were transfected with NC, miR-149-5p mimics, anti-NC, and miR-149-5p inhibitors in GM, and cell cycle phases were analyzed at 48 h after transfection by flow cytometry **(D,E)** and counted **(F,G)**. The results from each group are shown as the mean ± SEM of three independent replicates. Independent-samples *t*-tests were used for statistical analysis. Asterisks indicate significant differences. No asterisk means *P* > 0.05, *P < 0.05, and **P < 0.01.

We further examined the function of miR-149-5p in hair follicle stem cell proliferation by introducing NC, miR-149-5p mimics, anti-NC, or miR-149-5p inhibitors into stem cells. Hair follicle stem cells were transfected with miR-149-5p mimics or inhibitors and incubated for 96 h in GM. Stem cells were collected at 24-h intervals for RT-qPCR and western blotting against PCNA, CDK1, and CCND2, which are indicators of proliferation. miR-149-5p overexpression enhanced the mRNA expression of proliferation indicators (PCNA, CDK1, and CCND2) during hair follicle stem cell proliferation, but this change was not significant at some time points (such as PCNA mRNA expression in the mimics-treated group at 48 h, *P* > 0.05). By contrast, miR-149-5p inhibition downregulated PCNA, CDK1, and CCND2 mRNA levels during hair follicle stem cell proliferation, but this change did not reach a significant level in some periods (such as PCNA and CCND2 mRNA expression in the inhibitors-treated group at 24 h, *P* > 0.05) (Figures 4A–C). Similarly, PCNA, CDK1, and CCND2 protein levels were increased during hair follicle stem cell proliferation after miR-149-5p overexpression, whereas miR-149-5p inhibition decreased the protein expression of these proliferation indicators; however, this change was not significant at some time points (such as PCNA protein expression in the mimics-treated and inhibitors-treated groups at 48 h, *P* > 0.05). This decrease was accompanied by a decrease in the mRNA expression of proliferation indicators (PCNA, CDK1, and CCND2) during hair follicle stem cell proliferation (Figures 4D–G). Second, we examined the role of miR-149-5p in hair follicle stem cell proliferation with an EdU cell proliferation assay. We found that miR-149-5p overexpression (miR-149-5p mimics-treated group compared with NC counterparts) significantly increased the proportion of EdU-positive stem cells, but the proportion was notably decreased after miR-149-5p inhibition (miR-149-5p inhibitors treatments compared with anti-NC controls; *P* < 0.01) (Figures 5A–C). Moreover, the cell cycle assay further confirmed that miR-149-5p mimics promoted hair follicle stem cell proliferation, increased the number of hair follicle stem cells at S-phase (from 9.67% up to 12.36%, *P* < 0.05), and decreased the proportion of cells in G0/G1-phase (from 84.16% down to 82.53%, *P* > 0.05). Conversely, the results showed that after transfection with miR-149-5p inhibitors, the number of hair follicle stem cells in S-phase was reduced (from 14.06% down to 12.10%, *P* < 0.05), and the proportion of cells in G0/G1-phase was increased (from 79.32% down to 80.83%, *P* > 0.05) (Figures 5D–F). We also used a histogram to present the percentage of cells in each stage of the cell cycle, and only the difference in the percentage of cells in S-phase was significant (*P* < 0.05) after different treatments (Figure 5G). Taken together, these results indicated that miR-149-5p facilitates goat hair follicle stem cell proliferation.

### miR-149-5p Suppresses Goat Hair Follicle Stem Cell Apoptosis

**FIGURE 6:**
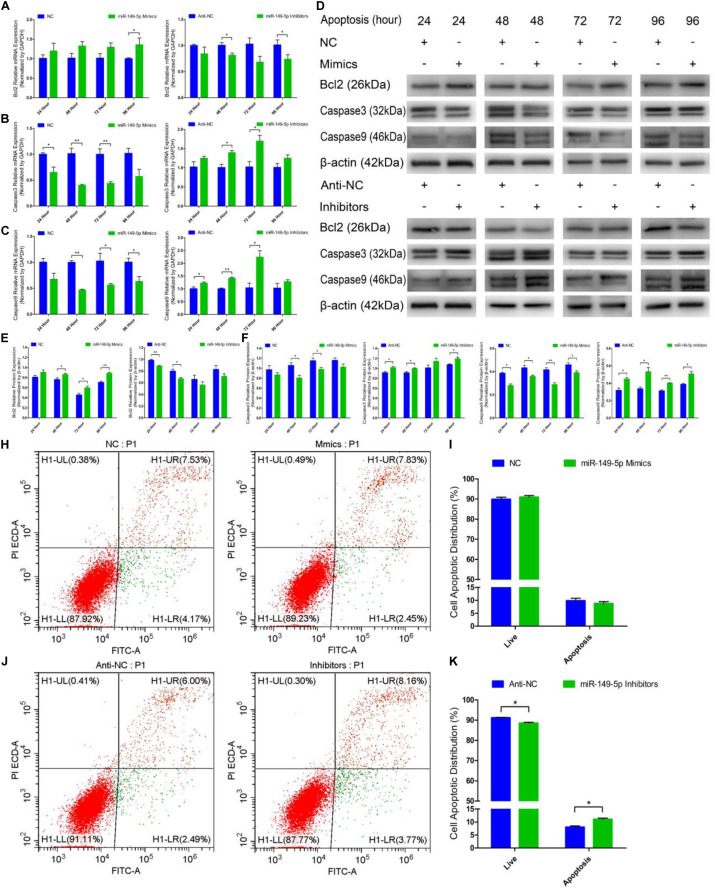
miR-149-5p suppresses goat hair follicle stem cell apoptosis. **(A)** mRNA expression of antiapoptotic gene (Bcl2) at 24 h, 48 h, 72 h, and 96 h after transfection with NC, miR-149-5p mimics, anti-NC, and miR-149-5p inhibitors in GM as determined by RT-qPCR. **(B,C)** mRNA expression of apoptosis marker genes (Caspase3 and Caspase9) at 24 h, 48 h, 72 h, and 96 h after transfection with NC, miR-149-5p mimics, anti-NC, and miR-149-5p inhibitors in GM as determined by RT-qPCR. **(D)** Protein expression of Bcl2 (1:1000 dilution), Caspase3 (1:1000 dilution), and Caspase9 (1:1000 dilution) was measured at 24 h, 48 h, 72 h, and 96 h after transfection with NC, miR-149-5p mimics, anti-NC, and miR-149-5p inhibitors in GM. β-actin (1:500 dilution) was used as an internal control. **(E)** Relative Bcl2 protein expression with NC, miR-149-5p mimics, anti-NC, and miR-149-5p inhibitors in GM at 24 h, 48 h, 72 h, and 96 h. **(F)** Relative Caspase3 protein expression with NC, miR-149-5p mimics, anti-NC, and miR-149-5p inhibitors in GM at 24 h, 48 h, 72 h, and 96 h. **(G)** Relative Caspase9 protein expression with NC, miR-149-5p mimics, anti-NC, and miR-149-5p inhibitors in GM at 24 h, 48 h, 72 h, and 96 h. **(H,I)** Hair follicle stem cells were transfected with NC or miR-149-5p mimics in GM, and cell apoptosis was analyzed 48 h after transfection by Annexin V-FITC/PI binding followed by flow cytometry. **(J,K)** Hair follicle stem cells were transfected with anti-NC and miR-149-5p inhibitors in GM, and cell apoptosis was analyzed 48 h after transfection by Annexin V-FITC/PI binding followed by flow cytometry. The results from each group are shown as the mean ± SEM of three independent replicates. Independent-samples *t*-tests were used for statistical analysis. Asterisks indicate significant differences. No asterisk means *P* > 0.05, *P < 0.05, and **P < 0.01.

Next, we examined the role of miR-149-5p in hair follicle stem cell apoptosis. We found that Bcl2 (an antiapoptotic gene) mRNA expression was increased in miR-149-5p mimics-treated cells compared with NC-treated cells at 24 h, 48 h, 72 h, and 96 h but was decreased in the miR-149-5p inhibitors-treated group at 24 h, 48 h, 72 h, and 96 h; however, these decreases were not significant at some time points (similar to Bcl2 mRNA expression in mimics-treated group at 24 h, 48 h, and 72 h, *P* > 0.05) (Figure 6A). The mRNA levels of Caspase3 and Caspase9, two marker-genes of apoptosis, were decreased in miR-149-5p mimics-treated cells compared with NC-treated cells at 24 h, 48 h, 72 h, and 96 h but increased in miR-149-5p inhibitors treated cells at 24 h, 48 h, 72 h, and 96 h; however, these changes were not significant at some time points (such as Caspase3 mRNA expression in inhibitors-treated group at 24 h and 96 h, *P* > 0.05) (Figures 6B,C). Similarly, Bcl2 protein levels were increased in the miR-149-5p mimics-treated group compared with the NC-treated group at 24 h, 48 h, 72 h, and 96 h and decreased in the miR-149-5p inhibitors-treated group at 24 h, 48 h, 72 h, and 96 h, but these differences were not significant at some time points (similar to the pattern of Bcl2 protein expression in the mimics-treated group at 24 h, *P* > 0.05) (Figures 6D,E). In contrast, Caspase3 and Caspase9 protein levels were reduced in the miR-149-5p mimics-treated group compared with the NC-treated group at 24 h, 48 h, 72 h, and 96 h and increased in the miR-149-5p inhibitors-treated group at 24 h, 48 h, 72 h, and 96 h; however, the differences did not reach a significant level at some time points (such as Caspase3 protein expression in the mimics-treated group at 24 h and 96 h, *P* > 0.05) (Figures 6D,F,G). The Annexin V-FITC/PI staining assay showed that miR-149-5p overexpression protected hair follicle stem cells from apoptosis and decreased the proportion of apoptotic cells (*P* > 0.05) (Figures 6H,I), while miR-149-5p inhibition induced apoptosis in hair follicle stem cells and increased the proportion of apoptotic cells (*P* < 0.05) (Figures 6J,K). Together, these results demonstrated that miR-149-5p suppresses goat hair follicle stem cell apoptosis.

### Ectopic miR-149-5p Expression Accelerates Goat Hair Follicle Stem Cell Proliferation and Inhibits Apoptosis

**FIGURE 7:**
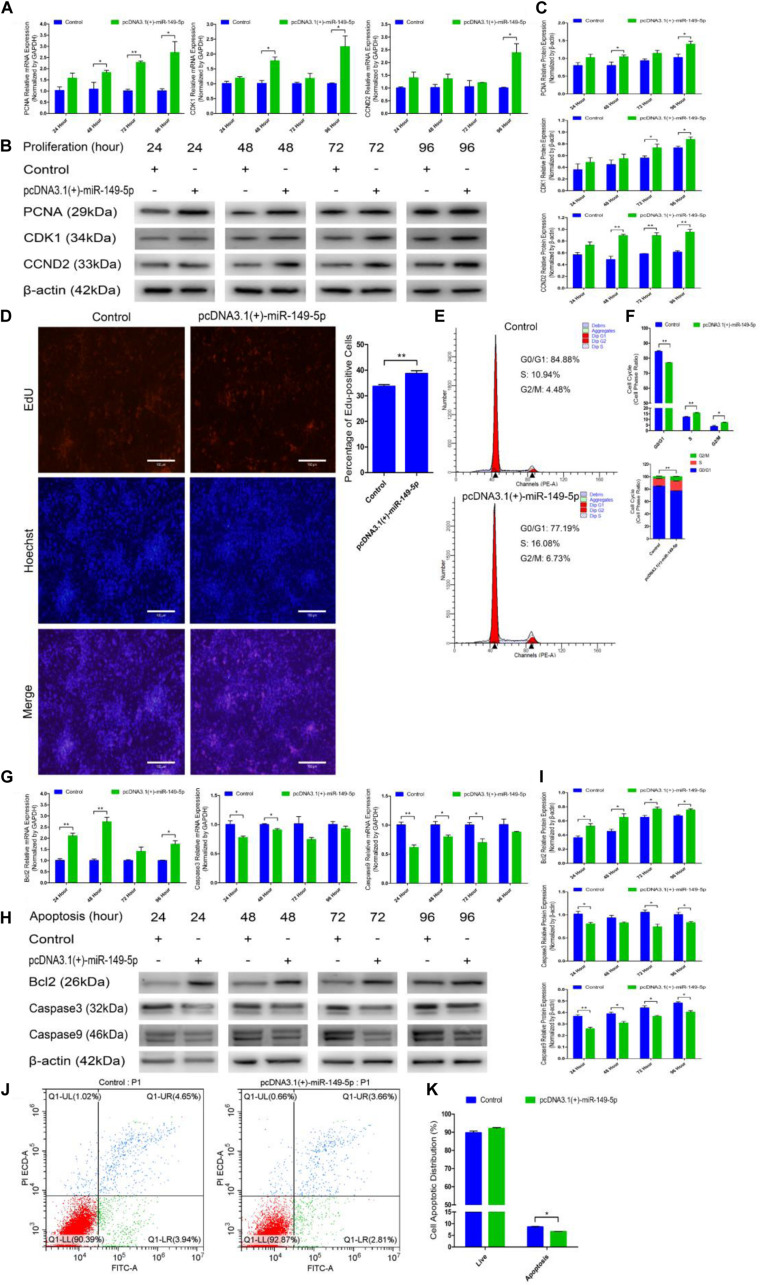
Ectopic miR-149-5p expression facilitates proliferation and inhibits apoptosis in goat hair follicle stem cells. **(A)** PCNA, CDK1, and CCND2 mRNA expression at 24 h, 48 h, 72 h, and 96 h after transfection with pcDNA3.1(+)-miR-149-5p in GM as determined by RT-qPCR. **(B,C)** Protein level of PCNA (1:1000 dilution), CDK1 (1:1000 dilution), and CCND2 (1:1000 dilution) were measured following overexpression of miR-149-5p with pcDNA3.1(+)-miR-149-5p in GM at 24 h, 48 h, 72 h, and 96 h. β-actin (1:500 dilution) was used as an internal control. **(D)** Representative images of the EdU assay of hair follicle stem cells at 24 h after transfection with pcDNA3.1(+)-miR-149-5p in GM. Bars, 100 μm. Hair follicle stem cells were transfected with pcDNA3.1(+)-miR-149-5p in GM, and cell phases were analyzed at 24 h after transfection by flow cytometry **(E)** and counted **(F)**. **(G)** mRNA expression of antiapoptotic gene (Bcl2) and apoptosis marker genes (Caspase3 and Caspase9) at 24 h, 48 h, 72 h, and 96 h after transfection with pcDNA3.1(+)-miR-149-5p in GM as determined by RT-qPCR. **(H,I)** Bcl2 (1:1000 dilution), Caspase3 (1:1000 dilution), and Caspase9 (1:1000 dilution) protein levels were measured following overexpression of miR-149-5p with pcDNA3.1(+)-miR-149-5p in GM at 24 h, 48 h, 72 h, and 96 h. β-actin (1:500 dilution) was used as an internal control. **(J,K)** Hair follicle stem cells were transfected with pcDNA3.1(+)-miR-149-5p in GM, and cell apoptosis was analyzed 48 h after transfection by Annexin V-FITC/PI binding followed by flow cytometry. The results from each group are shown as the mean ± SEM of three independent replicates. Independent-samples *t*-tests were used for statistical analysis. Asterisks indicate significant differences. No asterisk means *P* > 0.05, *P < 0.05, and **P < 0.01.

We constructed a miR-149-5p overexpression vector (pcDNA3.1[+]-miR-149-5p plasmid) to investigate the role of the miR-149-5p precursor in hair follicle stem cell proliferation and apoptosis. pcDNA3.1(+)-miR-149-5p was transfected into hair follicle stem cells to overexpress miR-149-5p, and the cells showed appreciably upregulated expression compared with that in the blank control or null-plasmid (pcDNA3.1[+])-treated groups, as detected by RT-qPCR (Supplementary Figure S2B). Next, pcDNA3.1(+)-miR-149-5p was transfected into hair follicle stem cells in GM, and the cells were collected at 24-h intervals. PCNA, CDK1, and CCND2 mRNA (Figure 7A) and protein (Figures 7B,C) levels were measured to determine the effect of miR-149-5p on hair follicle stem cell proliferation.

Compared with control cells, cells with miR-149-5p overexpression exhibited elevated levels of PCNA, CDK1, and CCND2 at 24 h, 48 h, 72 h, and 96 h, but these increases were not significant at some time points (such as PCNA mRNA expression in the overexpression vector treated group at 24 h, *P* > 0.05). Second, an EdU cell proliferation assay revealed that pcDNA3.1(+)-miR-149-5p clearly increased the proportion of EdU-positive stem cells (*P* < 0.01) (Figure 7D). Furthermore, the cell cycle assay confirmed that pcDNA3.1(+)-miR-149-5p promoted hair follicle stem cell proliferation, which was accompanied by a marked increase in the number of hair follicle stem cells in S-phase (from 10.94% up to 16.08%, *P* < 0.01) and a significant decrease in the proportion of cells in G0/G1-phase (from 84.88% down to 77.19%, *P* < 0.01) (Figures 7E,F).

pcDNA3.1(+)-miR-149-5p was also transfected into hair follicle stem cells in GM to assess the role of the miR-149-5p precursor in hair follicle stem cell apoptosis. The mRNA and protein expression levels of Bcl2, Caspase3, and Caspase9 were observed after transfection with the pcDNA3.1(+)-miR-149-5p plasmid in GM for 24 h, 48 h, 72 h, and 96 h. Compared with the control, the miR-149-5p precursor upregulated Bcl2 expression and downregulated Caspase3 and Caspase9 expression at 24 h, 48 h, 72 h, and 96 h in hair follicle stem cells, but these changes were not significant at some time points (such as Bcl2 mRNA expression in the overexpression vector treated group at 72 h, *P* > 0.05) (Figures 7G–I). Moreover, the Annexin V-FITC/PI staining assay showed that pcDNA3.1(+)-miR-149-5p protected hair follicle stem cells from apoptosis and decreased the proportion of apoptotic cells (*P* > 0.05) (Figures 7J,K). Taken together, these results revealed that the overexpression of miR-149-5p was able to promote hair follicle stem cell proliferation and inhibit stem cell apoptosis, which was consistent with the role of miR-149-5p mimics in both processes.

## Discussion

CMTM3, a member of the CMTM family, has been shown to play an important role in the development and progression of tumors (Zhong et al., 2006; Su et al., 2014) and to function as a regulator of AR transcriptional activity (Wang et al., 2008, 2018). Interestingly, our study confirmed that CMTM3 is a target gene of miR-149-5p by bioinformatic prediction and selection, RT-qPCR, western blotting, and dual-luciferase reporter gene assays. Androgen secretion could stimulate the formation of Type III superior-quality brush hair, and androgen can play its physiological function only after binding to AR. Hence, AR also plays a critical role in superior-quality brush hair traits. Androgens have also been identified to upregulate IGF-I expression and function as an important regulator of hair follicle growth (Philpott et al., 1994). In addition to these findings, we reported in a previous study that CMTM3 gene methylation promotes AR activity and upregulates the androgen hormone levels, which then results in the formation of superior-quality brush hair (Wang et al., 2018). In this work, CMTM3 expression was lower in the skin tissue of superior-quality brush hair goats than in those of the normal-quality brush hair goat but was not significantly different after analyzing three independent samples; furthermore, CMTM3 expression was decreased in cultured hair follicle stem cells in GM. In contrast, AR expression was higher in the skin tissues of superior-quality brush hair goats and increased in cultured hair follicle stem cells in GM. In addition, miR-149-5p expression was markedly higher in superior-quality brush hair goats than in normal-quality brush hair goats and was also upregulated in cultured hair follicle stem cells. These results suggested that miR-149-5p is involved in the regulation of hair follicle development and the formation of superior-quality brush hair. The formation of superior-quality brush hair is complex and involves hair follicle growth and development, which are driven by many biological and physiological processes (Stenn and Paus, 2001; Botchkarev and Sharov, 2004; Chen et al., 2012; Kretzschmar and Clevers, 2017). Therefore, based on these findings and bioinformatic analysis, we speculated that miR-149-5p influences CMTM3 expression, thereby increasing the expression of AR during the formation of superior-quality brush hair, which is similar to other miRNA/mRNA regulatory axes in skin and hair development (Botchkareva, 2012; Cai et al., 2013).

RT-qPCR and western blotting assays showed that AR mRNA and protein expression levels were increased after miR-149-5p overexpression and decreased when miR-149-5p was inhibited, suggesting that the miR-149-5p–CMTM3–AR axis is a critical regulator of hair follicle stem cell proliferation, apoptosis, and superior-quality brush hair traits. Moreover, we used the pDC316-mCMV-EGFP-CMTM3 vector (CMTM3-OE) and shRNA mediated CMTM3 experiments to investigate the effects of overexpressing or knocking down CMTM3, respectively, on the proliferation and apoptosis of hair follicle stem cells. We found that overexpressing CMTM3 decreased the number of hair follicle stem cells in S-phase and increased the number of cells in G0/G1-phase. However, knocking down CMTM3 conspicuously increased the proportion of cells in S-phase and decreased the proportion of cells in G0/G1-phase, which indicated that CMTM3 mainly plays an antiproliferative role during S-phase in goat hair follicle stem cell proliferation and functions contrary to miR-149-5p. These results were consistent with the regulatory role of CMTM3 in hepatocellular carcinoma cells (Li and Zhang, 2017) but in contrast to its function in gastric cancer cells (Lu et al., 2018). Annexin V-FITC/PI staining assays also showed that compared with the NC or sh-NC control, CMTM3 overexpression induced hair follicle stem cell apoptosis, whereas CMTM3 knockdown protected stem cells from apoptosis. RT-qPCR and western blotting assays showed that AR expression was decreased at both the mRNA and protein levels after CMTM3 overexpression, which is similar to the effect of miR-149-5p inhibitors on hair follicle stem cells. Conversely, CMTM3 knockdown increased the expression of AR, which is similar to the role of miR-149-5p mimics in stem cells. These results revealed that CMTM3 plays a negative role in hair follicle stem cell proliferation and apoptosis by influencing the expression levels of AR.

miR-149-5p is a member of the goat miR-149 family. Recent work has shown that miR-149-5p overexpression can activate Sirt1 activity and consequently protect the brain from resveratrol-induced ischemia by targeting p53, which offers a novel therapeutic approach during acute ischemic stroke (Teertam et al., 2020). The miR-149-5p precursor mitigates cell migration and invasion in renal cell carcinoma by targeting FOXM1 (Okato et al., 2017). However, miR-149-5p overexpression inhibits vascular smooth muscle cell proliferation, invasion, and migration by interacting with HDAC4 (Zhang et al., 2019), and it can function as a negative regulator of melanoma cell proliferation and cell survival and promote apoptosis by targeting LRIG2 (Chen et al., 2017), suggesting that miR-149-5p functions as a therapeutic molecule against melanoma. These studies have highlighted the different functions of miR-149-5p in cardiovascular disease and cancers. Our research indicated that miR-149-5p expression was upregulated in the skin tissues of superior-quality brush hair goats and in cultured hair follicle stem cells, similar to the expression trend of miR-128-3p in skin samples from angora rabbits (Zhao et al., 2019b). miR-149-5p overexpression promotes the expression of functional genes linked to proliferation (PCNA, CDK1, and CCND2) and represses the expression of apoptotic genes (Caspase3 and Caspase9) while upregulating the expression of the antiapoptotic gene Bcl2. By contrast, miR-149-5p inhibition suppresses the expression of functional genes related to proliferation (PCNA, CDK1, and CCND2) and antiapoptotic Bcl2 at the mRNA and protein level but accelerates the expression of proapoptotic genes (Caspase3 and Caspase9), which preliminarily illustrates that miR-149-5p upregulation can promote goat hair follicle stem cell proliferation and inhibit apoptosis. However, the mRNA levels of PCNA, CDK1, and CCND2 rapidly increased during the proliferation of hair follicle stem cells at 24 h after transfection with miR-149-5p mimics but decreased at 48 h, 72 h, and 96 h compared with the levels at 24 h. This phenomenon may be caused by the transient high expression of miR-149-5p after transfection with miR-149-5p mimics. In contrast to this phenomenon, the mRNA levels of Bcl2, Caspase3, and Caspase9 neither increased nor decreased quickly during the apoptosis of hair follicle stem cells at 24 h after miR-149-5p mimics treatment; however, Caspase3 and Caspase9 expression significantly decreased at 48 h, 72 h, and 96 h after miR-149-5p mimics treatment. These results could be explained by the fact that goat hair follicle stem cells are primarily engaged in proliferation for self-renewal purposes and show little apoptosis activity, and this trend could be maintained by miR-149-5p overexpression but weakened by miR-149-5p inhibition (Vasyliev et al., 2017; Lu et al., 2019). Additionally, EdU and cell cycle assays showed that miR-149-5p overexpression increased the proportion of EdU-positive cells, increased the number of hair follicle stem cells in S-phase, and decreased the proportion of cells in G0/G1-phase, whereas miR-149-5p inhibition decreased the proportion of EdU-positive cells, reduced the number of hair follicle stem cells in S-phase, and increased the proportion of the cells in G0/G1-phase. Interestingly, the trend of increased or decreased cells in G0/G1-phase was not significant in mimics-treated and inhibitors-treated groups. This result may be because miR-149-5p mainly plays a role in promoting proliferation at S-phase in goat hair follicle stem cell proliferation. In contrast to miR-149-5p, miR-134 could mediate S-phase arrest in human hepatocellular carcinoma cells (Ahn et al., 2019). Furthermore, Annexin V-FITC/PI staining assays showed that miR-149-5p overexpression protected hair follicle stem cells from apoptosis (decreased percentage of cells in early apoptosis), and miR-149-5p inhibition induced stem cell apoptosis (increased the rates of early and late apoptosis). These results indicate that miR-149-5p accelerates hair follicle stem cell proliferation and represses apoptosis, which is consistent with the role of miR-149-5p in pancreatic beta cells (Ruan et al., 2019). Interestingly, in contrast to our results, in renal cell carcinoma, the use of synthetic mimics to overexpress miR-149-5p can suppress cancer cell proliferation and migration but promote cancer cell apoptosis, with the apoptotic rate increasing from 1.89% (NC treatment) to 17.15% (miR-149-5p mimics treatment) (Jin et al., 2016). These findings indicate that the same miRNA plays diverse regulatory roles in different mammalian cells and tissues.

In the above results, the differences in some of the assays were not significant and only indicated increasing or decreasing trends, and we preliminarily characterized the relationship between CMTM3 and AR by using RT-qPCR and western blotting but not any other assays (such as coimmunoprecipitation assay) to further confirm the association between CMTM3 and AR. Therefore, elucidating the underlying mechanisms of superior-quality brush hair formation warrants further research.

## Conclusion

Our study determined that CMTM3 overexpression represses hair follicle stem cell proliferation and induces apoptosis; in contrast, CMTM3 knockdown accelerates hair follicle stem cell proliferation and protects stem cells from apoptosis. Moreover, AR expression was decreased after CMTM3 overexpression, which is consistent with the role of miR-149-5p inhibitors in hair follicle stem cells and was increased after CMTM3 knockdown, which is consistent with miR-149-5p mimics in stem cells.

In this study, we also showed that miR-149-5p is important for the formation of superior-quality brush hair traits and can promote goat hair follicle stem cell proliferation and suppress hair follicle stem cell apoptosis by inhibiting CMTM3 expression via a posttranscriptional mechanism. These results reveal a regulatory mechanism involving miR-149-5p, CMTM3, and AR, in which miR-149-5p controls goat hair follicle stem cell proliferation and apoptosis via the suppression of *CMTM3* and the upregulation of *AR*, and this mechanism further regulates the formation of superior-quality brush hair traits in Yangtze River Delta white goats.

## Data Availability Statement

All datasets generated for this study are included in the article/Supplementary Material.

## Ethics Statement

The animal study was reviewed and approved by Animal Care and Use Committee of Yangzhou University.

## Author Contributions

JW and YL: conceptualization. JW, YF, and JM: methodology. JW and JQ: software. LZ: validation. JW, JQ, YF, and JM: formal analysis. JW, CC, and HH: investigation. YL: resources. YW and DJ: data curation. YL: supervision and validation. JW: writing – original draft preparation. YL: writing – review and editing. All authors have read and agreed to the published version of this manuscript.

## Conflict of Interest

The authors declare that the research was conducted in the absence of any commercial or financial relationships that could be construed as a potential conflict of interest.

## Footnotes

1 https://www.ncbi.nlm.nih.gov/tools/primer-blast/index

2 http://c1.accurascience.com/miRecords

3 http://www.targetscan.org/-vert_72

4 http://www.mirbase.org

5 https://www.ncbi.nlm.nih.gov/

6 http://www.targetscan.org/vert_72

7 http://c1.accurascience.com/miReco-rds

8 https://david.ncifcrf.gov/

## Supplementary Material

Supplementary Figure 1 Morphological images and integrity checks in hair follicle stem cells. **(A,B)** Morphological images of hair follicle stem cells in GM harvested with a microscope. **(C)** Images of the integrity analysis of hair follicle stem cells with immunocytochemical staining (this result can be found in our published research paper in Gene Journal in 2019, Gene 698, 19–26).

Supplementary Figure 2 Efficiency of miR-149-5p oligos and its overexpression vector in goat hair follicle stem cells. **(A)** miR-149-5p expression 48 h after transfection with negative control (NC), miR-149-5p mimics (Mimics), single-stranded negative control (Anti-NC), and 2′-O-methylated oligonucleotides against miR-149-5p (Inhibitors) as determined by RT-qPCR. **(B)** miR-149-5p expression 48 h after transfection with pcDNA3.1(+) plasmid and pcDNA3.1(+)-miR-149-5p as determined by RT-qPCR. The results from each group are shown as the mean ± SEM of three independent replicates. Independent-samples *t*-tests were used for statistical analysis. Asterisks indicate significant differences. ^∗^*P* < 0.05, and ^∗∗^*P* < 0.01.

Supplementary Figure 3 Results of the preliminary dual-luciferase assay. **(A)** pcDNA3.1(+) or pcDNA3.1(+)-miR-365-3p was cotransfected with wild-type or mutant CMTM3 3′-UTR luciferase reporters in HEK293T cells. **(B)** pcDNA3.1(+) or pcDNA3.1(+)-miR-23a-3p was cotransfected with wild-type or mutant CMTM3 3′-UTR luciferase reporters in HEK293T cells. **(C)** pcDNA3.1(+) or pcDNA3.1(+)-miR-23b-3p was cotransfected with wild-type or mutant CMTM3 3′-UTR luciferase reporters in HEK293T cells. **(D)** pcDNA3.1(+) or pcDNA3.1(+)-miR-149-5p was cotransfected with wild-type or mutant CMTM3 3′-UTR luciferase reporters in HEK293T cells. The results from each group are shown as the mean ± SEM of three independent replicates. Independent-samples *t*-tests were used for statistical analysis. Asterisks indicate significant differences. No asterisk means *P* > 0.05, ^∗^*P* < 0.05.

## Abbreviations

ANOVA: analysis of variance
AR: androgen receptor
BMP signaling pathway: bone morphogenetic proteins signaling pathway
CDS: coding sequence
CKLF: chemokine-like factor
CMTM: CKLF-like marvel transmembrane domain-containing
CMTM3: CKLF-like marvel transmembrane domain-containing 3
DSG1: desmoglein 1
DMEM: Dulbecco’s modified eagle’s medium
EGFP: enhanced green fluorescent protein
EZH2: enhancer of zeste homolog 2
FBS: fetal bovine serum
FOXM1: forkhead box m1
GIT1: G protein-coupled receptor kinase interacting ArfGAP 1
HDAC4: histone deacetylase 4
HEK293T: human embryonic kidney 293T cells
IGF-I: insulin-like growth factors 1
IGF-IR: insulin-like growth factors 1 receptor
KATAPs: mammalian keratin-associated proteins
lncRNA SNHG8: long non-coding small nucleolar RNA host gene 8
MAPK: mitogen-activated protein kinase
RIPA: radio immunoprecipitation assay
PMSF: phenylmethanesulfonyl fluoride
SFPR2: the secreted frizzled-related protein 2
shRNA: short hairpin RNA
Sirt1: sirtuin1.
