# Supplementary figures and images for: miR-149-5p Regulates Goat Hair Follicle Stem Cell Proliferation and Apoptosis by Targeting the CMTM3/AR Axis During Superior-Quality Brush Hair Formation
Source: Front Genet. 2020 Nov 11;11:529757. doi: 10.3389/fgene.2020.529757 (PMC7686784; doi:10.3389/fgene.2020.529757)

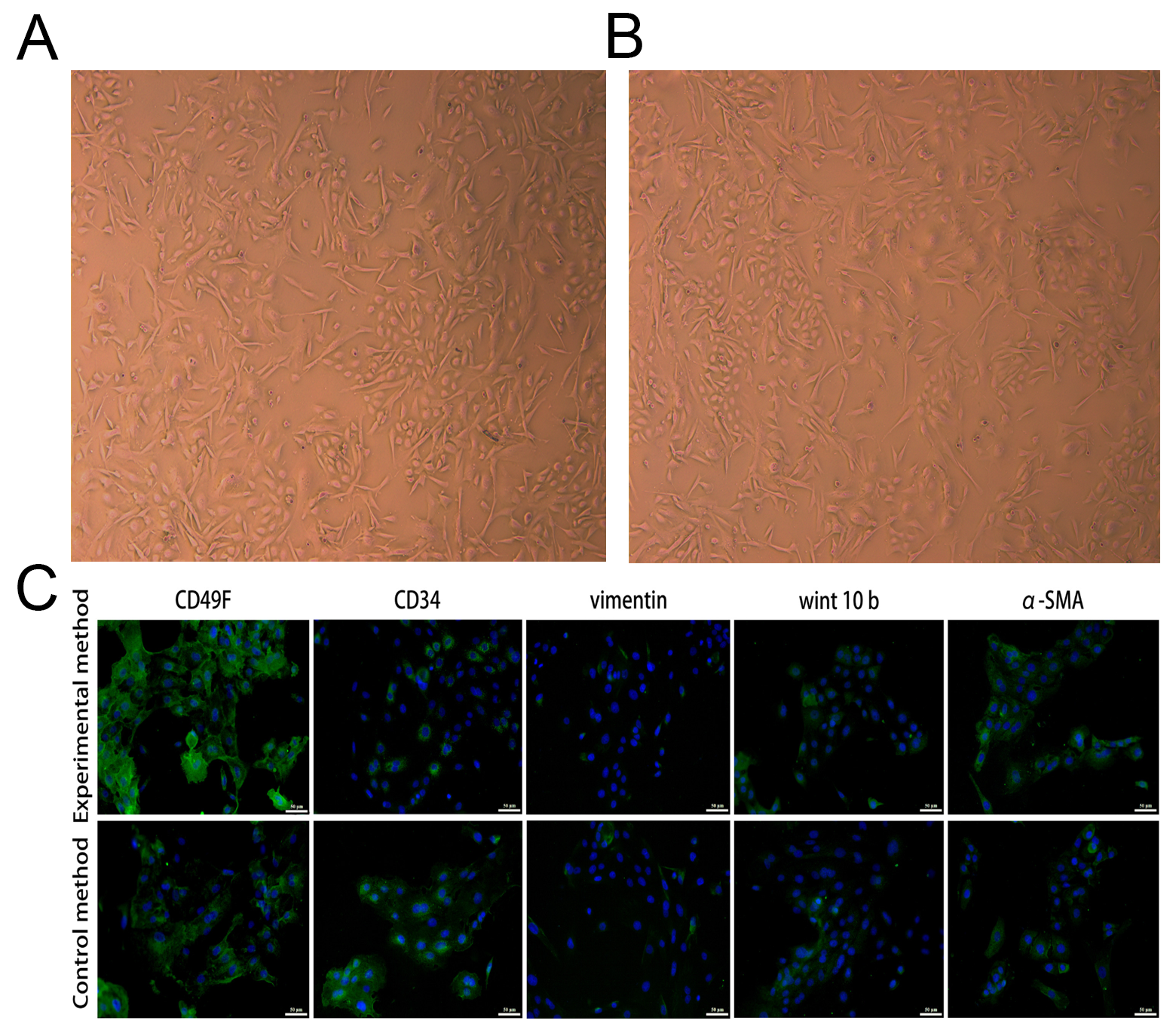

Supplement: Supplementary Figure 1 — Morphological images and integrity checks in hair follicle stem cells. (A,B) Morphological images of hair follicle stem cells in GM harvested with a microscope. (C) Images of the integrity analysis of hair follicle stem cells with immunocytochemical staining (this result can be found in our published research paper in Gene Journal in 2019, Gene 698, 19–26). [file Image_1.TIF]

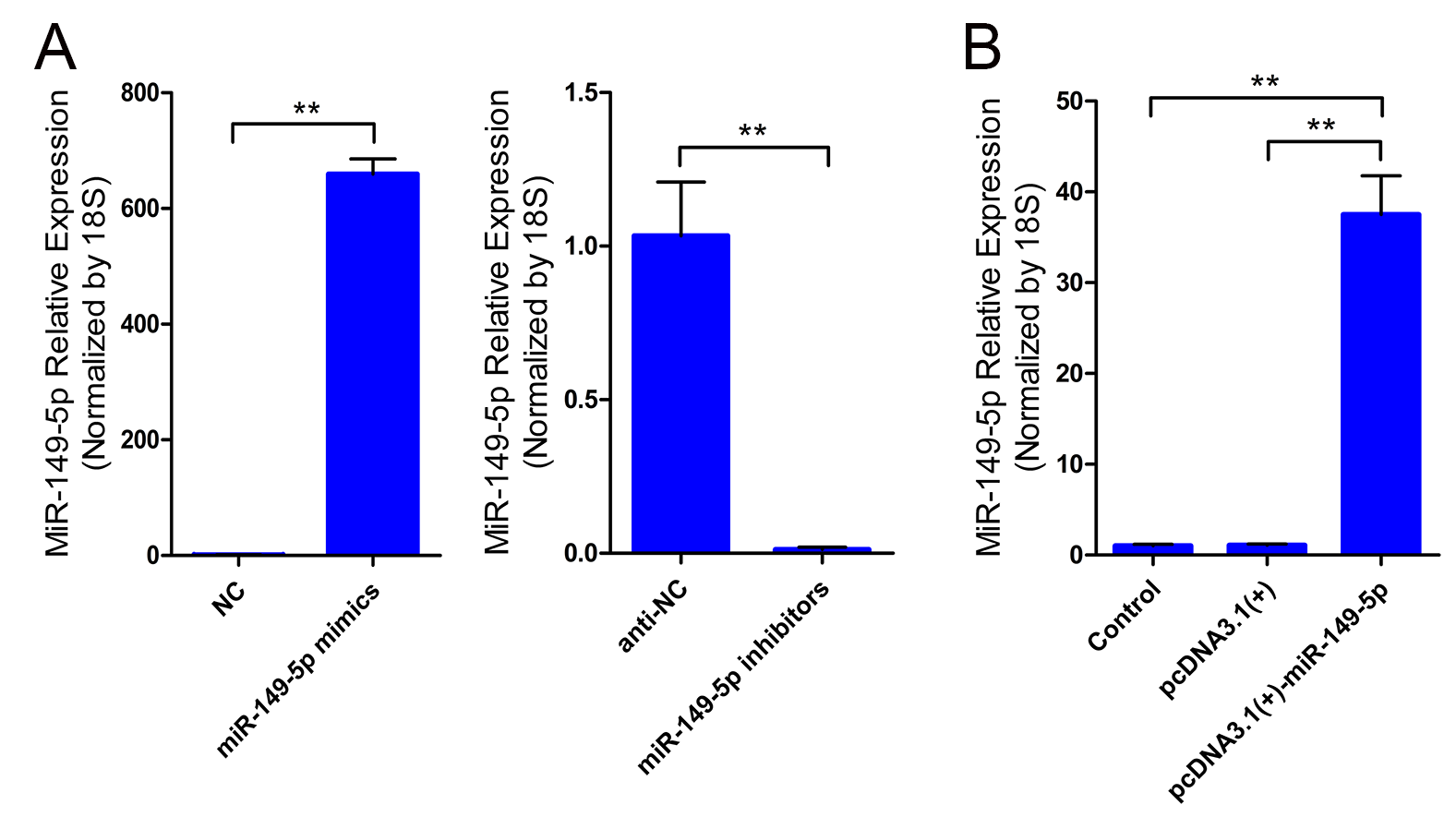

Supplement: Supplementary Figure 2 — Efficiency of miR-149-5p oligos and its overexpression vector in goat hair follicle stem cells. (A) miR-149-5p expression 48 h after transfection with negative control (NC), miR-149-5p mimics (Mimics), single-stranded negative control (Anti-NC), and 2′-O-methylated oligonucleotides against miR-149-5p (Inhibitors) as determined by RT-qPCR. (B) miR-149-5p expression 48 h after transfection with pcDNA3.1(+) plasmid and pcDNA3.1(+)-miR-149-5p as determined by RT-qPCR. The results from each group are shown as the mean ± SEM of three independent replicates. Independent-samples t-tests were used for statistical analysis. Asterisks indicate significant differences. ∗P < 0.05, and ∗∗P < 0.01. [file Image_2.TIF]
