# Supplementary material for: miR-149-5p Regulates Goat Hair Follicle Stem Cell Proliferation and Apoptosis by Targeting the CMTM3/AR Axis During Superior-Quality Brush Hair Formation
Source: Front Genet. 2020 Nov 11;11:529757. doi: 10.3389/fgene.2020.529757 (PMC7686784; doi:10.3389/fgene.2020.529757)
Supplement: Supplementary Figure 3 — Results of the preliminary dual-luciferase assay. (A) pcDNA3.1(+) or pcDNA3.1(+)-miR-365-3p was cotransfected with wild-type or mutant CMTM3 3′-UTR luciferase reporters in HEK293T cells. (B) pcDNA3.1(+) or pcDNA3.1(+)-miR-23a-3p was cotransfected with wild-type or mutant CMTM3 3′-UTR luciferase reporters in HEK293T cells. (C) pcDNA3.1(+) or pcDNA3.1(+)-miR-23b-3p was cotransfected with wild-type or mutant CMTM3 3′-UTR luciferase reporters in HEK293T cells. (D) pcDNA3.1(+) or pcDNA3.1(+)-miR-149-5p was cotransfected with wild-type or mutant CMTM3 3′-UTR luciferase reporters in HEK293T cells. The results from each group are shown as the mean ± SEM of three independent replicates. Independent-samples t-tests were used for statistical analysis. Asterisks indicate significant differences. No asterisk means P > 0.05, ∗P < 0.05. [file Data_Sheet_9.PDF]

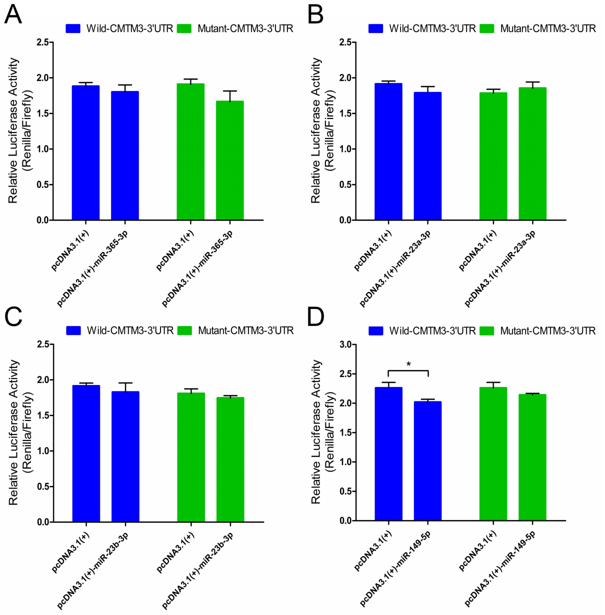
A
Wild-CMTM3-3'UTR
Mutant-CMTM3-3'UTR
Relative Luciferase Activity (Renilla/Firefly)
pcDNA3.1(+)
pcDNA3.1(+)-miR-365-3p
B
pcDNA3.1(+)-miR-23a-3p
C
pcDNA3.1(+)-miR-23b-3p
D
*
pcDNA3.1(+)-miR-149-5p
